# Perioperative Management of Coexisting Mast Cell Activation Syndrome and Malignant Hyperthermia Susceptibility: A Case Report and Novel Approach to Local Analgesia

**DOI:** 10.1155/cria/2750619

**Published:** 2026-10-27

**Authors:** Peter Francis Gearing, Thomas Nack, Dinuk Liyanage, Walston Reginald Martis, Michael Findlay

**Affiliations:** ^1^ Peter MacCallum Department of Oncology The University of Melbourne Melbourne 3010 Victoria Australia unimelb.edu.au; ^2^ Department of Plastic & Reconstructive Surgery Peter MacCallum Cancer Centre Melbourne 3000 Victoria Australia petermac.org; ^3^ Department of Anaesthesia Perioperative and Pain Medicine Peter MacCallum Cancer Centre Melbourne 3000 Victoria Australia petermac.org; ^4^ Department of Anaesthesia Perioperative and Pain Medicine Monash Health Clayton 3168 Victoria Australia monashhealth.org; ^5^ Department of Critical Care The University of Melbourne Melbourne 3010 Victoria Australia unimelb.edu.au; ^6^ Department of Surgery Monash University, Clayton Melbourne 3800 Australia

**Keywords:** malignant hyperthermia, mast cell activation syndrome, perioperative anaphylaxis, procedural sedation

## Abstract

The coexistence of mast cell activation syndrome (MCAS) and malignant hyperthermia (MH) presents a significant perioperative challenge due to combined risks of anaphylaxis and anaesthesia‐related hypermetabolic crisis. We describe the successful anaesthetic management of a man in his 60 s with confirmed MCAS and a strong family history of MH who underwent excision of multiple malignant cutaneous lesions. A structured perioperative strategy incorporating immunological optimisation with omalizumab, strict trigger avoidance, dexmedetomidine and remifentanil procedural sedation, prophylactic corticosteroids and a controlled low‐dose adrenaline infusion enabled safe completion of surgery without complication. In the absence of tolerated local anaesthetic agents, dilute adrenaline infiltration was used as an adjunct to improve procedural tolerability. This case highlights the importance of multidisciplinary planning and individualised perioperative strategy in patients with high‐risk perioperative profiles.

## 1. Introduction

Mast cell activation syndrome (MCAS) is a heterogeneous disorder characterised by inappropriate mast cell mediator release, resulting in episodic multisystem symptoms including urticaria, bronchospasm, hypotension and anaphylaxis [1, 2]. Reactions may be IgE‐mediated or non‐IgE‐mediated. Perioperative exposure to medications, antiseptics, mechanical stimuli and physiological stress may precipitate severe reactions [3]. Malignant hyperthermia (MH) is an inherited pharmacogenetic disorder of skeletal muscle calcium regulation, most commonly associated with pathogenic variants in the ryanodine receptor (*RYR1*) gene. It is transmitted in an autosomal dominant pattern and may precipitate a life‐threatening hypermetabolic crisis following exposure to volatile anaesthetics or depolarising neuromuscular blocking agents [4] and is associated with increased immune system activity [5]. The coexistence of MCAS and MH susceptibility is rarely reported and created a uniquely high‐risk perioperative profile, significantly restricting anaesthetic options and necessitating meticulous multidisciplinary planning. This report presents the difficulties encountered in the case of MCAS and familial MH susceptibility, written according to CARE case report guidelines.

## 2. Case Presentation

A man in his 60 s presented for elective excision of six malignant cutaneous lesions in the setting of confirmed MCAS. He had a strong family history of MH (father and grandfather; father died intraoperatively with a clinical diagnosis of MH) but had not undergone formal genetic testing nor in vitro contracture testing. His medical history included IgA multiple myeloma (previous stem cell transplant 10 years prior), obstructive sleep apnoea (on Continuous Positive Airway Pressure [CPAP] therapy), chronic renal impairment, prior pulmonary embolism, heart failure with reduced ejection fraction (45%) and steroid‐induced insulin‐dependent diabetes mellitus. His regular medications included amlodipine (10 mg mane), apixaban (5 mg twice daily), atorvastatin (40 mg nocte), cetirizine (20 mg mane), furosemide (80 mg mane, 40 mg midday), glyceryl trinitrate (5 mg/24 h patch), metformin (1000 mg twice daily), metoprolol (95 mg mane), montelukast (10 mg/3 month), omalizumab (300 mg/fortnight), prednisolone (2.5 mg daily) and spironolactone (50 mg mane).

He had documented severe hypersensitivity reactions to multiple agents including chlorhexidine and povidone‐iodine, local anaesthetics (bupivacaine, ropivacaine, lidocaine and prilocaine), opioids (morphine, buprenorphine and oxycodone), induction agents (propofol, midazolam, ketamine–tested as monotherapy agents in previous anaesthetics), several antibiotic classes, NSAIDs, contrast media, heparin, immunoglobulins and adhesive tapes and latex did not induce a reaction. Several prior reactions were perioperative anaphylactic episodes with airway involvement requiring intensive care admissions (seven ICU admissions in the last 10 years). Formal allergen testing had not been undertaken. Tryptase levels were within normal limits.

The diagnosis of MCAS was confirmed by the immunology service according to internationally validated criteria [6]. His symptoms were optimised with omalizumab and maintenance prednisolone. Surgery was scheduled within 7 days of omalizumab administration to align with peak therapeutic effect and was placed first on the operating list to facilitate extended monitoring. A multidisciplinary planning meeting involving anaesthesia, surgery and immunology was undertaken. The theatre and anaesthetic machine were prepared according to MH‐safe protocols with removal of volatile agents and vaporisers and dantrolene was immediately available [4]. Dantrolene hypersensitivity had not been previously tested for.

Premedication comprised intravenous hydrocortisone 200 mg and oral cetirizine 10 mg. In light of contraindications to volatile anaesthetic agents, propofol and benzodiazepines, a sedation‐based technique was selected. Procedural sedation was achieved using remifentanil administered via target‐controlled infusion (Minto model; effect‐site concentration 3 ng/mL) in combination with dexmedetomidine (0.5–1.0 μg/kg/h); both remifentanil and dexmedetomidine had been previously tolerated. Depth of anaesthesia was monitored clinically and titrated to signs of pain (and verbal scores, i.e., numeric rating scale, when awake). Oxygenation was maintained using transnasal humidified rapid‐insufflation ventilatory exchange (THRIVE). Continuous invasive haemodynamic monitoring was instituted. A prophylactic low‐dose adrenaline infusion (2 μg/min) was commenced to support haemodynamic stability and to facilitate immediate escalation to therapeutic intravenous adrenaline dosing should clinical features of anaphylaxis develop. No adrenaline‐related adverse effects were noted (including clinically significant tachycardia, arrhythmia or myocardial ischaemia).

Given antiseptic intolerance, the surgical field was prepared with sterile saline. A solution of 1:100,000 adrenaline in saline was infiltrated locally for haemostasis. The adrenaline solution was also injected according to surface anatomical landmarks, to target locoregional sensory nerves. Lesions were excised sequentially by two surgeons and closed with nontriclosan‐coated Monocryl sutures to avoid additional allergen exposure. Smaller lesions were excised first; larger lesions were excised after a minimum of 25 min to allow for peak vasoconstriction. The procedure lasted 60 min and was well tolerated. An adrenaline nebuliser was administered prophylactically at completion. The patient was monitored in the postanaesthesia care unit (PACU) and admitted for overnight observation. He was discharged on postoperative day one without complication. A detailed perioperative plan was documented for future procedures.

## 3. Discussion

This case demonstrates the complexity of managing coexisting MCAS and MH susceptibility. In MCAS, perioperative reactions may be triggered by medications, antiseptics, mechanical stimulation, or stress. Avoidance of known triggers, pretreatment with antihistamines and corticosteroids and biologic therapy such as omalizumab may reduce the risk of severe mediator release [7]. Omalizumab, an anti‐IgE monoclonal antibody, may decrease mast cell reactivity when continued perioperatively. MH susceptibility mandates strict avoidance of volatile anaesthetics and depolarising neuromuscular blocking agents [8]. While total intravenous anaesthesia (TIVA) is generally considered safe in MH, this patient’s documented hypersensitivity to propofol, ketamine and midazolam significantly constrained available options.

A sedation‐based approach avoided both general anaesthesia and neuromuscular blockade. Remifentanil was selected as it offers both sedation and analgesia, provides rapid titratability and has a low reported incidence of hypersensitivity reactions [9]. It was combined with dexmedetomidine to offer anxiolytic effects without respiratory depression and to reduce the total remifentanil dose in the setting of OSA [10]. The addition of a low‐dose adrenaline infusion provided haemodynamic support and immediate escalation capability should anaphylaxis occur. Importantly, contingency planning included MH‐safe machine preparation, availability of dantrolene, invasive monitoring and postoperative observation.

In the absence of tolerated local anaesthetic agents, a novel, pragmatic strategy was employed with the aim of improving procedural tolerability. A dilute solution of adrenaline (1:100,000) in saline was infiltrated subcutaneously along the planned incision lines and in proximity to the relevant cutaneous nerve territories based on surface anatomical landmarks. The primary intent was haemostasis; however, we hypothesised that an adjunctive reduction in nociceptive input could be observed by targeting sensory neural vasa nervorum [11]. This mechanism by which adrenaline alone may modulate pain is not well established, and it is unlikely to provide true local anaesthesia (in the absence of sodium channel blockade). However, local vasoconstriction may decrease tissue perfusion and neural signalling and limit the delivery of inflammatory mediators to peripheral nociceptors.

Importantly, the effect of adrenaline infiltration observed in this case was likely to be modest and insufficient as a sole analgesic modality for general procedures. In our patient, it was used in conjunction with systemic analgesia (remifentanil) and sedation (dexmedetomidine), which together provided tolerable procedural conditions, and likely outweighed the effect of adrenaline alone. The delayed onset of maximal vasoconstriction was accounted for by staging excision, allowing approximately 20 min between infiltration and more stimulating components of the procedure. The patient was very satisfied with his procedure and has since undergone two further skin cancer excision procedures with the same periprocedural anaesthetic strategy.

To our knowledge, the use of dilute adrenaline alone for locoregional infiltration in the setting of true local anaesthetic intolerance has not been described in the surgical literature. A single dental case report describes the use of diphenhydramine with adrenaline as an alternative local anaesthetic in patients with hypersensitivity to amide agents [12]. Our approach differs in that no alternative sodium channel‐blocking agent was used. While unlikely to replace conventional local anaesthesia, our technique could be considered in highly selected cases where standard anaesthesia options are contraindicated. Further studies would be required to determine reproducibility, efficacy and safety, although the rarity of true local anaesthetic anaphylaxis may limit prospective evaluation. In the absence of this research, it should be used with caution given the risks of systemic adrenaline absorption leading to cardiovascular complications and delayed wound healing.

## 4. Conclusion

In patients with coexisting MCAS and MH susceptibility, meticulous multidisciplinary planning and individualised pharmacological strategy are essential. When both volatile agents and standard intravenous induction agents are contraindicated, dexmedetomidine‐remifentanil sedation with controlled adrenaline support may provide a safe alternative. When local anaesthetics are contraindicated, we present a pragmatic approach to infiltration of dilute adrenaline to target sensory nerves. Although single‐case experience limits generalisability, this report demonstrates that structured perioperative planning can enable safe surgical management in patients with high‐risk profiles.

## Funding

The authors have not declared a specific grant for this research from any funding agency in the public, commercial or not‐for‐profit sectors. Open access publishing facilitated by The University of Melbourne, as part of the Wiley ‐ The University of Melbourne agreement via the Council of Australasian University Librarians.

## Ethics Statement

Peter MacCallum Cancer Centre Ethics Committee waives ethics submissions for case reports.

## Consent

Written consent was obtained and is retained by the institution.

## Conflicts of Interest

The authors declare no conflicts of interest.

## Data Availability

The data that support the findings of this study are available on request from the corresponding author.
